# Perspective: Beyond the cherry on top: positioning nutrition where it belongs

**DOI:** 10.1177/17449871251362662

**Published:** 2025-09-02

**Authors:** Francesca Tabacchi

**Affiliations:** Lecturer in Dietetics and Nutrition, Oxford Brookes University, Oxford, UK

My connection to cancer care is not merely academic: it is deeply personal and professional. It is shaped by years of being at patients’ bedsides, often at vulnerable moments, trying to help them nourish themselves while they endured, or prepared for, treatment. I worked in the NHS for seven years, specialising as oncology dietitian before moving into education and research.

As a dietitian, what drew me to cancer care was the impact that my role could have. I saw patients regain strength, tolerate chemotherapy better, or simply find a bit more comfort in an otherwise chaotic time. I saw how amazing a dietetic service in a free healthcare system can be. But I also saw what happened when that system was not fast enough. Malnutrition affects up to 80% of people with cancer depending on tumour site and stage. Some patients – even one is too many – were referred only when they were already malnourished or depleted. Others were eager to receive nutritional information but were dismissed or left waiting due to system bottlenecks. It broke my heart to see this happening, and to think how preventable much of it was. These feelings and the questions they raised became the driver of my PhD research, for which I interviewed both cancer patients and specialist oncology nurses: What is not working in our system? Why is nutrition not integrated into care? And what can we realistically do about it in practice?

These are not abstract questions, and they are not just for dietitians. They are questions for all staff but especially for nurses, who are central to cancer care in a way few others are.

Nurses are often the first and the key contact in a patient’s journey. They are trusted and present when treatment plans are being made, when symptoms flare and when families need reassurance. In theory, and increasingly in policy, they are also expected to screen for malnutrition, provide basic first-line dietary advice, and identify when a referral to a dietitian is needed.

This is positive because nurses work holistically, seeing the whole person. But it is also risky because nurses are stretched across more responsibilities than ever. And because nutrition is not always part of core training or specialistic training.

In many services, there simply are not enough dietitians to work with every patient who needs support. So, we rely on nurses, which is often not only successful but also fragile, because it depends on individual nurses’ knowledge and available time.

This is why I am writing this perspective piece, to invite conversation, collaboration, and clarity about what could realistically change in practice. And perhaps, to ensure nutrition has a stronger, earlier place at the table, before patients lose the strength to sit at it.

It’s important to be clear: dietitians are the professionals clinically responsible for assessing and managing nutrition. Their expertise is essential, particularly for patients with complex or high-risk needs. With limited staffing, dietitians are often forced to prioritise those in more severe states of malnutrition, meaning others may only be seen if referred early on by other professionals.

## When nutrition is missed, everyone bears the consequences

Malnutrition after a cancer diagnosis has clear consequences: more complications, longer hospital stays, and lower quality of life (Ryan et al., 2016). Despite this, nutrition is often overlooked. Screening is inconsistent, referrals delayed, documentation patchy and conversations about food are frequently vague or absent (Lorton et al., 2020). One of my participants said:

When I asked about nutrition, my oncologist just rolled his eyes and said: ‘It doesn’t make any difference.’

Patients interviewed in my research consistently voiced frustration and a sense of neglect when it came to nutrition support. Many felt dismissed or left to navigate their needs alone, often at a time when guidance was most critical:

Having that conversation at the beginning would’ve made me feel more supported - not just like a number being processed through a machine.

Malnourished patients often experience worse side effects, lower tolerance to treatments like chemotherapy or radiotherapy, and in the worst cases, are forced to pause or stop treatment entirely. The emotional toll of untreated malnutrition extends beyond the patient. Families and loved ones often bear the distress of watching someone struggle with feeding, leading to anxiety, helplessness, and burnout, all of which further diminish quality of life and can even interfere with treatment decisions and adherence (Van Cutsem and Arends, 2005).

But patients are not the only ones who pay the price. Nurses and other frontline staff often absorb the consequences, emotionally and practically, when nutrition is missed. They spend more time managing preventable complications, fielding urgent concerns and trying to support patients in crisis. The wider system pays too. Late-stage malnutrition is not only harder to reverse, but it is also more resource-intensive. Longer admissions, unplanned readmissions and poorer recovery all strain services that are already stretched. Waiting until patients are in crisis is not just inefficient, it is unethical.

Timing is everything in nutrition. Proactive, early support is empowering; it gives patients tools and confidence to care for themselves while preparing for treatment. Many healthcare professionals lack training in nutrition or feel too overwhelmed to address it adequately. Some, consciously or not, still perceive it as secondary to treatment, missing its critical role in improving outcomes and supporting patient resilience (Arends et al., 2017).

A woman who participated to a patient involvement workshop during my PhD expressed it perfectly:

Nutrition is seen as a cherry on top – but it should be part of the cake.

That single comment captures the fundamental misunderstanding embedded in many cancer care systems: the idea that nutrition is a luxury, not a necessity. Too often, patients are told vague and contradictory things about nutrition. But that advice, while well-meaning, is rarely helpful, especially when patients are anxious, confused, or desperately seeking ways to support their recovery.

As one participant in my research put it:

I was just advised to eat what I want. I was told that by nurses, by my radiographer, and I think my consultant probably said it as well. They were all just like, ‘eat . . . what you want.’ I did ask about supplements, but they were very wishy-washy about what I could or couldn’t take.

The marginalisation of nutrition in cancer care reflects a system that too often reacts rather than prepares. But the tide is turning. Growing evidence now supports a proactive, integrated approach: prehabilitation, that places nutrition, physical activity, and psychological support at the heart of preparing patients for treatment.

## Prehabilitation: preparing the person holistically

Prehabilitation, which is the supporting of patients *before* treatment begins, is becoming more recognised, but is not yet standard. It focuses on improving physical, psychological, and nutritional status before cancer treatment, from chemotherapy to surgery. It is an opportunity to strengthen the body and the overall well-being so that patients are in the best position possible to cope with what is ahead (Cancer Support, n.d.). In some cases, it may also include support for finances, lifestyle changes, or complementary therapies.

The participants in my study embraced the concept wholeheartedly:

I’d have liked to be in the best possible shape for surgery. I’d have wanted that support – physically and nutritionally.

Prehab isn’t just about exercise. It’s about nutrition and mental strength too.

Despite this, nutritional prehabilitation is rarely standard practice. In the United Kingdom, there is growing interest, with trials and small services emerging, but delivery is still patchy. Patients are not routinely referred to prehab programmes unless they are part of a study or a pilot pathway. Guidelines and national strategies advocate for offering prehab to all cancer patients but in reality only a few benefit from it. This inconsistency creates inequity and missed opportunity.

Evidence shows that prehabilitation improves treatment tolerance, reduces complications and hospital admissions and enhances quality of life. It also gives patients a sense of action: something many crave after a difficult diagnosis (Lee et al., 2016).

One patient I interviewed described this poignantly:

I think I would have appreciated it. Yeah, I’d have wanted to be in the best possible physical position I could be in to be able to have the surgery and recuperate, you know? And I think all those things feed in.

What is striking is that many patients intuitively understand the value of prehabilitation, even if it was not offered to them. They seek out charities, online resources, or try to prepare by themselves. But it is not their responsibility alone.

It just seems odd . . . with heart disease or diabetes, they tell you how important diet is. But with cancer, it’s like ‘oh just eat what you want.’ That doesn’t make sense to me at all.

Scientific evidence supports this: improved tolerance to treatment, fewer complications, and better recovery (Muscaritoli et al., 2021). So why does not this happen more often? Barriers are systemic. Prehabilitation takes time, staffing and coordination. It requires multiple allied health professionals (AHPs) to be involved early and working together. Funding is inconsistent and awareness varies across departments. Training and structured referral pathways are underdeveloped. Many services still rely on short-term projects or have limited access to it. Nutrition is sometimes included but not always.

Yet, the potential is huge. This is where nurses come in, not as an extra burden for them, but as partners in early identification and empowerment. With the right support, nurses can signpost patients to reliable information or charities and advocate for earlier referrals. Their involvement sends a powerful signal that nutrition matters.

Prehabilitation is not necessarily about doing more, it is about doing what we already do earlier and with more coordination. Nurses are already present in these early stages. With the right tools and collaboration, they can help make prehabilitation a standard part of the cancer care cake, not just a nice occasional cherry on top.

It would’ve helped, especially before surgery. I think if I had that information before, I would’ve felt much more in control.

## Empowering, not overwhelming: what nurses can do – and already do

From my research, patients did not expect nurses to deliver full dietary plans. They mostly wanted acknowledgement and safe information. A nurse said:

‘If you don’t eat, how will you recover?’ That stuck with me.

Nurses themselves, in my interviews, were open and willing to contribute to nutritional care. Many saw nutrition as part of their role already but would have liked more training and confidence. Most were happy to refer but noted time pressures, unclear pathways or lack of contact with dietitians.

I’m ashamed to say I don’t know what protocols are in place.

When dietitians had time to deliver informal teaching, nurses felt more informed and connected. And when referral systems were quick and simple, action followed. Other barriers included lack of IT support, unclear guidance on supplements or special diets, and fear of overwhelming the small dietetic services. Still, there was a consistent theme: nurses were aware of the need. They just needed systems that made it easier to respond to that need.

There are all these amazing scientists doing this work . . . but it doesn’t come to us. It doesn’t make it into practice.

The learning from my research was clear: nutrition support in cancer care needs to start earlier, be more visible and be delivered through better communication and collaboration. Across interviews with patients and oncology professionals, certain patterns emerged again and again. When nutritional care worked, it was personalised, empowering and practical. Patients described feeling supported when advice was tailored, not vague or dismissive.

The solutions to improve nutritional care are often small and practical:

- Documenting weight changes and making them visible in notes or handovers
- Asking one or two nutritional questions during routine care
- Validating patient nutritional concerns
- Having reliable nutrition leaflets or website links ready
- Simply acknowledging the role of nutrition in preparation and recovery

These things do not require extra appointments or systemic changes. But they can shift the culture around nutrition, helping patients feel that food, weight, appetite, and well-being belong in cancer conversations. Small, consistent actions can shift the culture. But to make this sustainable, the system must support them.

At the service level, we need:

- Bite-sized Continuous Professional Development (CPD) and training sessions designed for real-world nursing contexts
- Electronic health records that automatically flag unintentional weight loss or nutrition-related risks
- Simple, integrated referral pathways
- Routine, meaningful contact between nursing and dietetic teams
- Clear, practical protocols that leave room for local adaptation

At the system level, the priorities are broader and goals are longer-term:

- Universal access to prehabilitation, not just in trial settings
- Greater investment in dietetic staffing
- Protected time for nutritional assessment and interventions
- Rigorous research into the economic case for early nutritional support

But systems do not change on data alone. They change when professionals across disciplines reinforce the same message: nutrition matters. When patients hear that message consistently from nurses, doctors, dietitians, and radiographers; it builds trust, reduces confusion, and increases the chance that they will take action.

As one nurse put it:

The boundaries are blurring more. We’re working together more collaboratively, but I think there’s still a way to go.

This collaboration is key. Nutrition – it is a shared responsibility. But nurses often spend the most time with patients, which makes their role critical. With better tools, training, and system-level support, nurses can help embed nutrition not as an add-on, but as a core part of cancer care.

We are at a turning point. The evidence is clear, patient voices are getting louder and nursing insight is pushing the field forward. Now we need action. Nutrition must be embedded into every stage of the cancer pathway, from diagnosis to treatment to recovery. When we treat it as foundational rather than optional, we do not just improve outcomes, we dignify care. And in doing so, nurses can lead the way in transforming how we care for the whole person, not just for the disease.

## Practice going forward

Dietitians hold clinical responsibility, but their capacity is often stretched. The future of oncology nutrition lies in shared effort: professionals bringing their unique strengths, working across boundaries and always centring the patient. The closeness nurses have to patients makes them powerful advocates and connectors. When nurses acknowledge nutrition, offer a leaflet, ask a question or simply validate a patient’s concern, it makes a real difference. And patients notice it. Many participants in my research described moments where nursing staff brought warmth, clarity and practical support, often when others had not.

So when I was diagnosed the gynae nurse I saw said it's very important to keep your weight up. So I’m not losing, not losing weight.’

I’m just like a rabbit in the headlights but maybe with the nurse, maybe, then have a conversation with the nurse. It’s a bit more gentle, you know.

Eating is something we all do every day: paying attention to it is a simple talk that helps humanise cancer care. In a healthcare system that can sometimes be perceived by people living with cancer as oppressive and purely medical, looking after people’s nutrition is a simple gesture that patients will remember fondly, and that can make a difference not only in terms of treatment but also in the way patients experience treatment.
